# Effect of Reinforcement with Short Carbon Fibers on the Friction and Wear Resistance of Additively Manufactured PA12

**DOI:** 10.3390/polym15153187

**Published:** 2023-07-27

**Authors:** Abdelrasoul Gadelmoula, Saleh Ahmed Aldahash

**Affiliations:** 1 Department of Mechanical and Industrial Engineering, College of Engineering, Majmaah University, Al-Majmaah 11952, Saudi Arabia; saldahash@mu.edu.sa; 2 Department of Mechanical Design and Production Engineering, Faculty of Engineering, Assiut University, Assiut 71515, Egypt

**Keywords:** carbon fiber, polyamide 12, selective laser sintering, friction and wear, frictional noise

## Abstract

Reinforcing thermoplastic materials for additive manufacturing with either short, long, and continuous fibers or micro/nanoparticles is a sound means to enhance the mechanical/tribological properties of functional 3D printed objects. However, despite the fact that reinforced thermoplastics are being used extensively in modern applications, little data are found in open literature regarding the effect of such reinforcements on the friction and wear characteristics of additively manufactured objects. Therefore, this article presents a comparative study that aims to investigate the friction and wear behavior of carbon fiber-reinforced polyamide 12 (CF-PA12) as compared to pure polyamide 12 (PA12). The test specimens were prepared by selective laser sintering (SLS) at five different build orientations and examined using a pin-on-disc tribometer in dry sliding mode. The coefficient of friction (COF), interface temperature, friction-induced noise, and specific wear rate were measured. Scanning electron microscopy (SEM) was used to inspect the tribo-surfaces. The results revealed that both the COF and contact temperature of CF-PA12 are orientation-independent and are lower than those of pure PA12. Also, it was found that, compared with pure PA12, CF-PA12 has 25% smaller COF and 15–40% higher wear resistance. Further, the SEM of tribo-surfaces showed that adhesive wear dominates the surface of pure PA12, while both adhesive and abrasive wear patterns coexist in CF-PA12. Moreover, fiber crushing and thinning were observed, and this, under some circumstances, can result in a considerable increase in frictional noise.

## 1. Introduction

Recently, additive manufacturing (AM) techniques have evolved to the extent that they are being used to produce functional parts. Selective laser sintering (SLS) is a popular AM technique in which selected areas of the thermoplastic powder layer are sintered using laser energy, thus allowing objects to be manufactured layer-by-layer. The SLS process is unique in that it does not require any object supporting structure since the object being manufactured is supported by the unsintered powder in the build chamber. However, the currently available thermoplastic powders do not meet all the required properties of commercial parts [1]. Therefore, fillers in the form of fibers or particulates are introduced to enhance the mechanical and/or tribological properties of laser-sintered objects. Hence, short, long, and continuous carbon fibers (CFs), glass fibers, or thermoplastic fibers have been included as potential reinforcements. Likewise, graphite, glass beads, MoS_2_, PTFE, and other particulates have been used as fillers. Among previous fillers, carbon fibers are known for their high stiffness, high tensile strength, chemical and wear resistance, high thermal conductivity and low thermal expansion, and low weight. Further, as the properties of objects prepared by the SLS process are orientation-dependent [1,2], reinforcing the SLS thermoplastic powders with short CFs enhances the homogeneity of manufactured parts significantly [3,4], and improves the wear resistance of carbon fiber-reinforced composites [5,6]. Accordingly, CFs have been used as a reinforcement for thermoplastic composites used in automotive, motorsport, aerospace, and military industries [6,7,8]. However, although reinforcing thermoplastics with CFs effectively increases its tensile strength and decreases the coefficient of friction (COF), frictional heating, and specific wear rate, it was found that the maximum elongation at break was reduced significantly [8,9,10,11], which modulates the friction and wear behavior of CF-reinforced composites.

Among the different types of polyamide (PA), PA12 is known to be the most suitable material for SLS because of its favorable characteristics of low melting temperature, very low hygroscopicity, high crystallinity, and excellent dimensional stability. Further, laser-sintered PA12 parts are characterized by its biocompatibility, high tensile strength, high impact resistance, excellent chemical resistance, electrical insulation, and the ability to withstand sudden drops in working temperatures. Moreover, in dry sliding conditions, PA12 was proven to have a low coefficient of friction, good wear resistance, and good resistance to stress cracking. Therefore, PA12 finds increasing applications in automotive industries, oil/gas pipelines, sports equipment, photovoltaics industry (PV), and electrical appliances. Accordingly, to further increase the applicability of PA12 in demanding industries, carbon fibers were introduced as a filler to enhance the mechanical as well as the tribological properties of laser-sintered parts based on this material [12,13]. Currently, end-use parts of carbon fiber-reinforced polyamide 12 (CF-PA12) composite are commercially available. The CF-PA12 is fine black thermoplastic composite known for its lightweight, electric conductivity, extreme stiffness, high strength and hardness, and prominent thermal stability; indeed, good bonding between CFs and the PA12 matrix in the SLS process was observed, which imparts such superior tensile strength of the CF-PA12 composite [3,9]. Hence, because of its high strength and light weight, CF-PA12 is being used in many applications such as aerospace, automobiles, motor sports, airframes, body armors, and housings. Consequently, direct interaction of CF-PA12 with mating surfaces in such applications is inevitable. So far, the mechanical properties of laser-sintered CF-PA12 composite was the focus of most published works. However, despite the favorable properties of CF-PA12, comparatively little has been reported regarding the tribological properties of SLS objects based on this composite. Therefore, this work is devoted to investigating the friction and wear characteristics of the CF-PA12 composite prepared using the SLS process as compared to those of pure PA12. For this purpose, CF-PA12 as well as pure PA12 specimens manufactured along five different build orientations will be examined using pin-on-disc tribometer at dry sliding conditions against a stainless disc. The coefficient of friction (COF), contact temperature, friction-induced noise, and specific wear rates will be measured. Finally, scanning electron microscopy (SEM) will be used to inspect the worn tribo-surfaces. Combined with the relevant results in the literature, the experimental findings from this study can help the designers in selecting the proper SLS build orientation of CF-PA12 tribo-surface for minimum coefficient of friction, maximum wear resistance, and low level of friction-induced noise.

## 2. Materials and Methods

### 2.1. Materials

**Figure 1:**
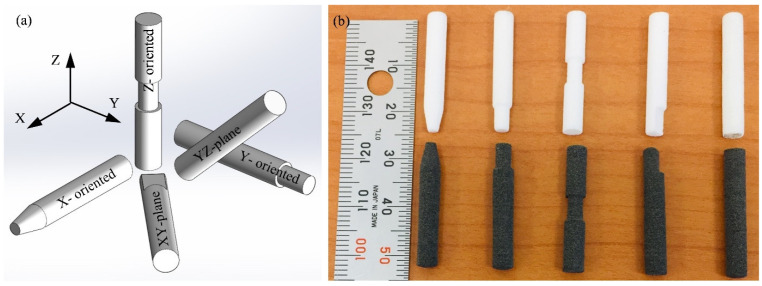
(**a**) Schematics of specimens’ orientation in SLS build chamber; (**b**) manufactured specimens, PA12 (upper) and CF-PA12 (lower).

The SLS powders used for the manufacturing of test specimens are PA2200 (PA12) and CarbonMide (CF-PA12) supplied by EOS GmbH (Düsseldorf, Germany). As the properties of SLS objects are orientation-dependent, a set of five test specimens was manufactured of pure PA12 and another set was manufactured of the CF-PA12 composite. The test specimens are oriented in the build chamber of the SLS machine as shown in Figure 1a. The pin-shaped test specimen is 4 mm diameter and 25 mm long. The SLS fabrication parameters of both materials are given in Table 1. Micro cuts of different geometries were introduced to facilitate the sorting of the specimens when extracted from the build chamber (see Figure 1b). The test specimens are built along the positive Z-axis, and a set of three test specimens is manufactured along each orientation. To ensure dry sliding conditions, tribo-surfaces of the specimens were kept away from dust and oily substances.

The fabrication parameters in the SLS process (i.e., laser power, scanning speed, scan spacing, and layer thickness) have a significant effect on the physical properties of fabricated objects [14,15,16], hence the values given in Table 1 are those set by the manufacturer to maximize object density and reduce the porosity of fabricated parts.

### 2.2. Experimental Methods

**Figure 2:**
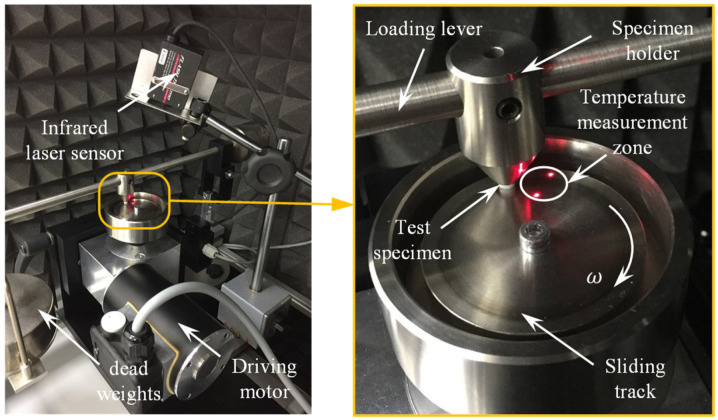
Experimental test-rig using pin-on-disc tribometer [17].

The pin-shaped CF-PA12 and pure PA12 specimens were examined using the pin-on-disc tribometer at which the specimen was inserted in a pin holder and loaded against a rotating stainless steel disc by means of an end-loaded lever, as shown in Figure 2. To maintain dry sliding conditions, the active surface of the disc was cleaned with 99.9% ethanol to remove any dirt, oily substance, or adsorbed matter, and the experiments were conducted at low humidity environment (less than 10%). The approximate interface temperature was measured using an infrared laser sensor (FT-H30, KEYENCE, Itasca, IL, USA) along with a digital amplifier unit (FT-50AW, KEYENCE, Itasca, IL, USA). The sensor detects the disc temperature over a circular area of 6 mm diameter in the vicinity of pin/disc contact zone and after 0.05 s of detachment, then an Arduino Uno microcontroller board was used to handle the output signal. To exclude the effect of polymer transfer film, each experiment was conducted using a new stainless steel disc. Additionally, the discs were manufactured using the same cutting conditions to yield similar surface roughness, and the measured (Ra) value along the radial direction was around 0.36 ± 0.05 microns. To measure the friction-induced noise, the experimental test-rig was arranged inside a semi-anechoic chamber and the noise level was measured using a digital sound level meter (GM1357, Benetech co., Shenzhen, China) with 30–130 dB measurement range and ±1.5 dB of accuracy, then an Arduino Uno microcontroller (SMD R3, Arduino.cc, Ivrea, Italy) was used to manage the output signal.

The friction force was measured using a double-bending beam force sensor along with NI controller (CompactDAQ, NI, Austin, TX, USA), and a moving average function with a Labview code was used to smooth out the signal and record the COF every 0.5 s. Also, an Arduino Uno microcontroller board (SMD R3, Arduino.cc, Ivrea, Italy) was used to manage the output signals of both the infrared laser sensor and the digital sound level meter. Finally, scanning electron microscopy (SEM) (SNE-4500M Plus, Sec Co., Suwon, South Korea) was used for characterizing the tribo-surfaces of examined specimens.

## 3. Results and Discussion

To evaluate the effects of introducing carbon fibers (CF) as a reinforcement on the friction and wear characteristics of carbon fiber-reinforced polyamide 12 (CF-PA12) composite prepared using the SLS technique, pin-shaped samples of CF-PA12 and pure PA12 were examined with the aid of a pin-on-disc tribometer in dry sliding mode. The results presented in this section were obtained at a normal load of 50 N, disc rotation speed of 120 rpm, and for a duration of 45 min. Table 2 shows the applied experimental conditions. 

Since the diameter of the pin-shaped specimens is 4 mm, the apparent contact pressure was about 4 MPa; however, the real contact pressure is much higher than the apparent contact pressure since the real contact area is 10–30% of apparent contact area [18]. The present experimental conditions were chosen to investigate the tribological characteristics of both materials under harsh operating conditions of high contact pressure and low sliding speed.

The results presented in this section address the comparison between CF-PA12 and pure PA12 regarding the coefficient of friction, contact temperature, friction-induced noise, specific wear rate, and SEM characteristics of worn tribo-surface. The main objective is to get a clear insight into the effect of introducing short carbon fibers as reinforcement of the PA12 matrix in SLS objects.

### 3.1. Coefficient of Friction (COF)

**Figure 3:**
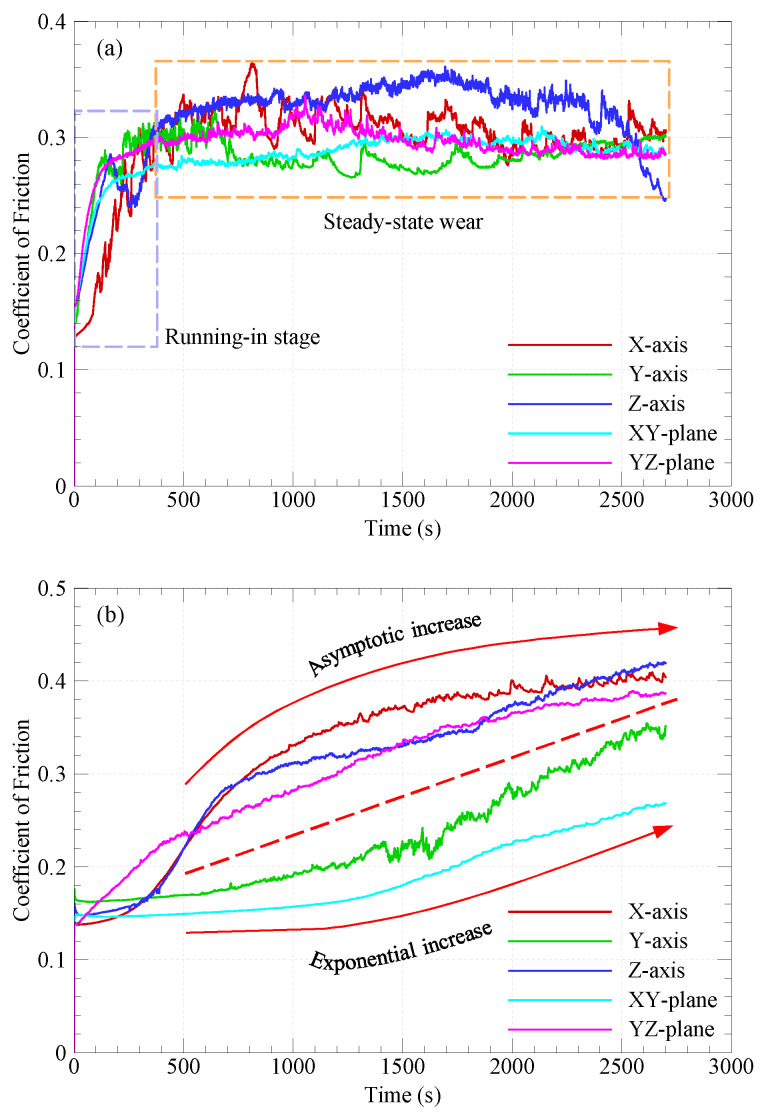
Variations of friction coefficient with sliding time. (**a**) CF-PA12; (**b**) pure PA12.

A moving average function was used to average the measured friction forces over a period of 0.5 s, and then the average friction force is divided by the applied normal load (50 N) to obtain the average COF over this time span. The variations of the COF with sliding time for the CF-PA12 composite and pure PA12 are shown in Figure 3 for different build orientations. The most notable finding from Figure 3 is that the variation pattern of the COF for CF-PA12 specimens built along different orientations (see Figure 3a) features two distinct stages; a running-in stage, which lasts for few minutes, followed by a steady-state zone. On the other hand, the variations of the COF for pure PA12 specimens (see Figure 3b) is characterized by the presence of two patterns of increase: asymptotic increase (with X-, Z-, and YZ-oriented specimens), and exponential increase (with Y- and XY-oriented specimens). Another finding that stands out from Figure 3 is that the COF of CF-PA12 specimens is about 25% lower than that of pure PA12 (considering the asymptotic value). The rapid increase in the COF at the running-in stage of CF-PA12 specimens is attributed to the frequent formation and shearing out of adhesive junctions at the sliding interface since it was reported that most frictional resistance is associated with adhesion at the interface [19,20].

The contradictory evolutions of the COF between CF-PA12 and pure PA12 specimens can be explained from the viewpoint of adhesion resistance in dry sliding mode as follows: in the case of CF-PA12 specimens, the fibers are encapsulated with sintered PA12 because of the good bonding characteristics between carbon fibers and the PA12 matrix [9]. As a result of dry sliding over the steel disc, adhesion at the interface occurs, which results in the rapid removal of PA12 film from carbon fibers, putting carbon fibers into direct contact with the disc surface and, consequently, the load-bearing capacity of the CF-PA12 composite increases and adhesive wear declines, thus causing a relatively stable COF over a wide region of the steady-state zone. Meanwhile, for pure PA12 specimens (without load-bearing reinforcements), the combination of normal loading and dry sliding conditions causes a gradual growth in the real contact area, which, in turn, accelerates the formation of adhesive junctions at the interface, causing the COF to increase with such an exponential manner, as shown in Figure 3b. Another interesting finding from Figure 3 is that, contrarily to pure PA12, the COF of CF-PA12 specimens is almost orientation-independent; this can be attributed to the fact that reinforcement of the PA12 matrix with short carbon fibers enhances the homogeneity of the resulting composite, and this result agrees well with previous studies [3,4]. Moreover, the results from Figure 3 show that the Z-oriented specimen exhibits the highest COF compared to the specimens built along other orientations throughout the steady-state region; however, its COF drops sharply after about 40 min of sliding. This behavior may be attributed to the solid lubrication effect of carbon fibers debris, as will be explained later in detail. Meanwhile, the high-frequency oscillations in the COF are owing to the corresponding oscillations in the frictional resistance introduced by irregularly aligned short CFs in the CF-PA12 surface layer [21].

### 3.2. Interface Temperature

**Figure 4:**
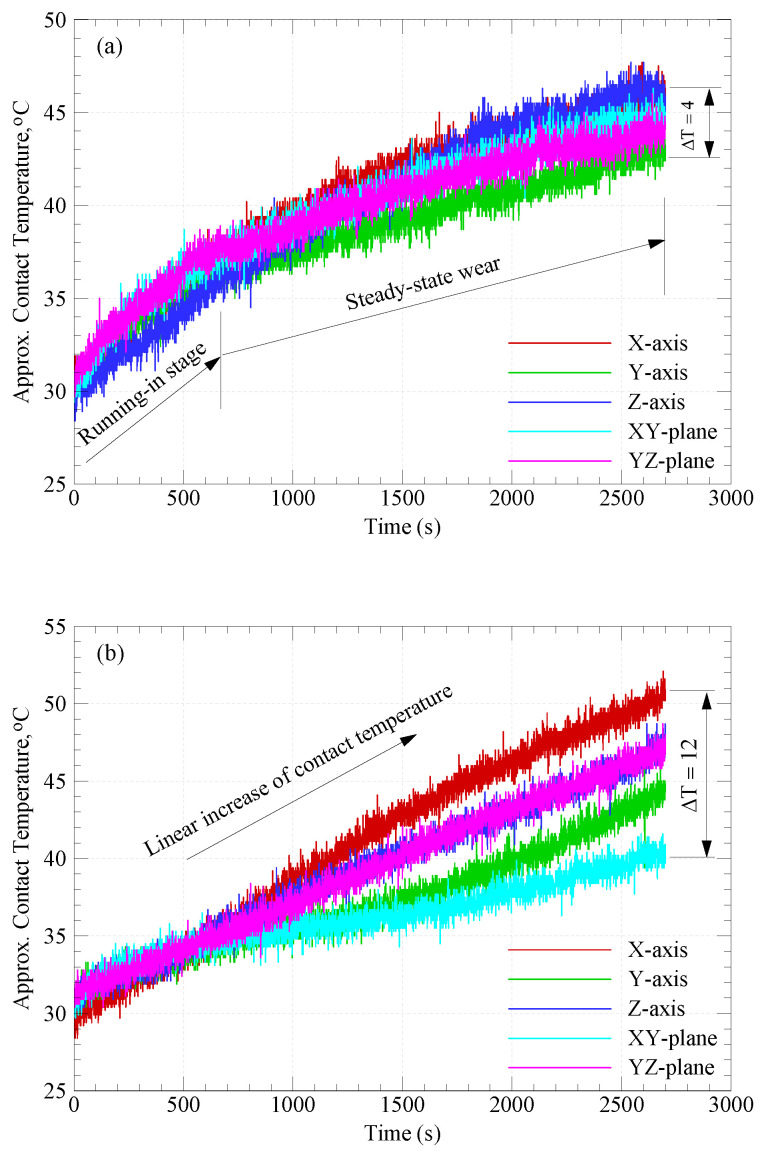
Variations of approximate contact temperature with sliding time. (**a**) CF-PA12; (**b**) pure PA12.

To overcome the frictional resistance to sliding (i.e., resistance to surface and sub-surface elastic/plastic deformations, and resistance to shearing of adhesive junctions at the interface), an input work must be spent. Once sliding occurs, most of the spent work is transformed into frictional heat [19,22]. Figure 4 shows the variations of the approximate contact temperature with sliding time for CF-PA12 and pure PA12 specimens built along different orientations. The comparison reveals that the evolution of the CF-PA12 contact temperature (shown in Figure 4a) is consistent with its COF (shown in Figure 3a); the sharp increase of CF-PA12 contact temperature in the running-in stage is a direct result of the sharp increase in the COF during the same period. However, the CF-PA12 contact temperature increases in a sluggish manner during the steady-state wear stage because of frictional heat buildup in the disc. Additionally, the results from Figure 4a show that the contact temperature of CF-PA12 specimens built along the five different orientations are very close and the difference after 45 min of dry sliding is only about 4 °C. in contrast to CF-PA12, the contact temperature of pure PA12 shows a linear increase with sliding time. Furthermore, the results from Figure 4b suggest that the contact temperature of pure PA12, similarly to its COF, is orientation-dependent and the difference between contact temperatures becomes pronounced as the sliding time increases (the difference is about 12 °C after 45 min of sliding). 

Indeed, the most important result from Figure 4 is that the contact temperatures of CF-PA12 specimens are lower than those of pure PA12 at all build orientations. There are two likely explanations for such difference between contact temperatures of CF-PA12 and pure PA12: the first explanation is that the higher thermal conductivity of carbon fibers, opposite to PA12, allows the frictional heating to be transferred in-part to the specimens, thus reducing the temperature gradient at the interface [7,23]. The second explanation is that introducing carbon fibers as a reinforcement reduces PA12 adhesion to the disc surface by supporting most of the normal load and acts to remove the transfer film from the disc surface [23]. Moreover, carbon fiber crushing enhances the lubricity at the disc–specimen interface, thus reducing the COF and, consequently, the contact temperature.

### 3.3. Friction-Induced Noise

**Figure 5:**
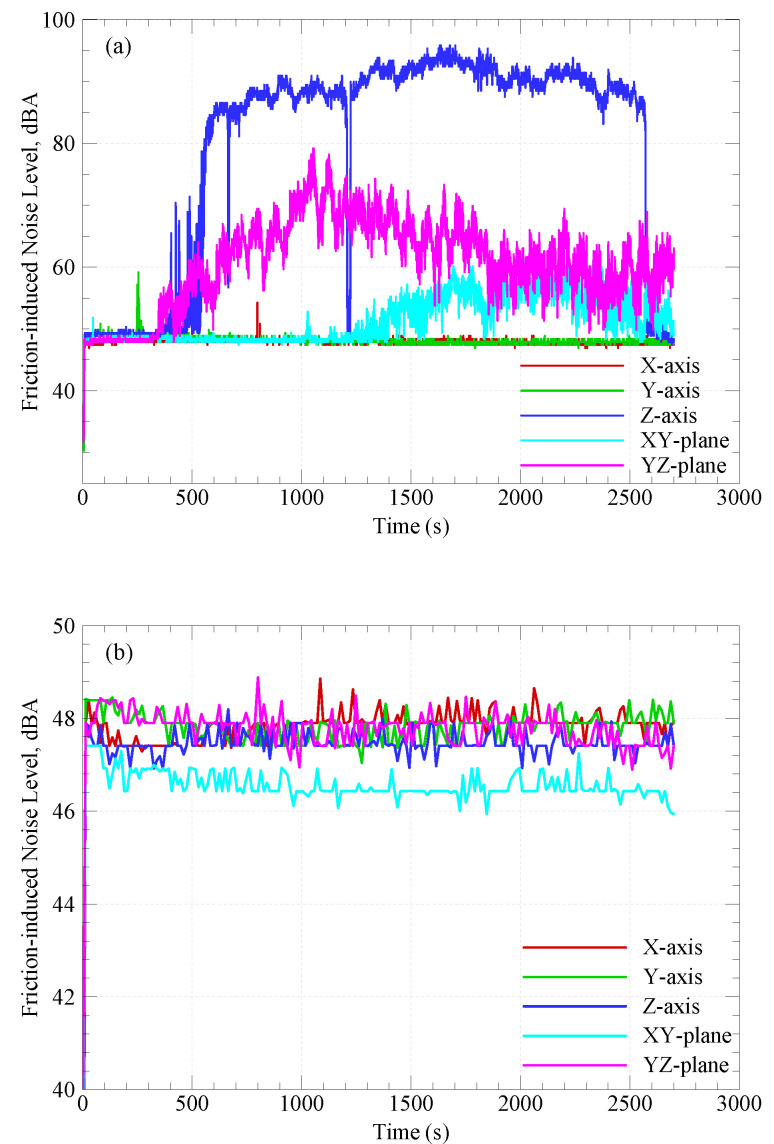
Variations of friction-induced noise with sliding time. (**a**) CF-PA12; (**b**) pure PA12.

Frictional noise is attributed to the continuous formation and shearing out of adhesive junctions at the interface [17]; hence, reducing the COF results in reducing the friction-induced noise [24]. Figure 5 shows the variation of friction-induced noise with sliding time for CF-PA12 and pure PA12 specimens at different build orientations where the background noise in the experimentation site was 35–37 dBA. A comparison of the results plotted in Figure 5a,b reveals that pure PA12 specimens have superior frictional noise characteristics compared to that of CF-PA12 counterparts. Also, the Z-oriented CF-PA12 specimen exhibits the highest frictional noise among all specimens. A close look at the evolution of frictional noise with sliding time shows that the reinforcement of PA12 with carbon fibers alters both the noise level and frequency after a while (see Z-, XY-, and YZ-orientated CF-PA12 specimens). 

**Figure 6:**
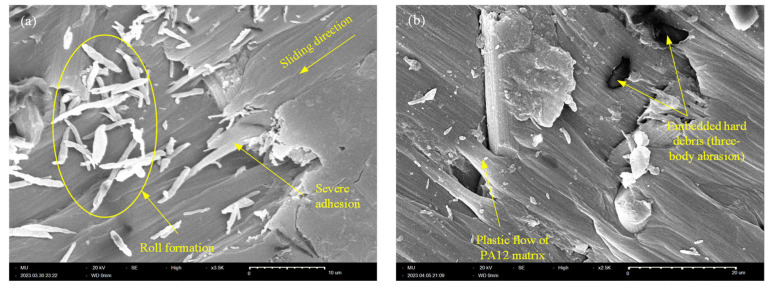
SEM of Z-oriented CF-PA12. (**a**) Adhesion combined with roll formation; (**b**) three-body abrasion.

The continuous removal of PA12 during the running-in stage brings the carbon fibers into direct contact with the rotating disc. As carbon fiber is a high-stiffness amorphous material, asperities interlocking combined with abrasion resistance result in such increases in the frictional noise level. Eventually, pulled-out graphite particles from carbon fibers act to lubricate the interface, thus reducing the COF and, consequently, the noise level [25]. The striking increase of the frictional noise level of Z-oriented CF-PA12 is attributed to the severe adhesion at the interface (see Figure 6); such strong adhesion causes localized tearing and/or delamination of the PA12 matrix. This results in the separation of PA12 debris that either rolls between the interacting surfaces creating what is called "roll formation" or temporarily attaches to the counter surface as a three-body abrasion (see Figure 6a). Moreover, because of the PA12 matrix softening that is associated with the rapid formation and shearing out of adhesive junctions, hard debris are embedded easily in the PA12 matrix, as shown in Figure 6b; as a result, the embedded hard debris causes severe abrasion of the counter surface and extremely elevated levels of frictional noise.

The remarkable low noise level of pure PA12 specimens is attributed to the fact that adhesion, which dominates the interface in the case of PA12 dry sliding over hard surfaces, results in the formation of a transfer film on the disc surface, which, in turn, reduces the further transfer of PA12, and thus maintaining such a uniform pattern of frictional noise. Furthermore, the results show that X- and Y-oriented specimens of CF-PA12 and pure PA12 have a comparable noise level; this is likely attributed to the alignment of carbon fibers in sintered layers, as it was reported that 50–60% of carbon fibers are aligned along the X-axis, 30–40% along the Y-axis, and 5–15% along the Z-axis [26].

### 3.4. Specific Wear Rate

**Figure 7:**
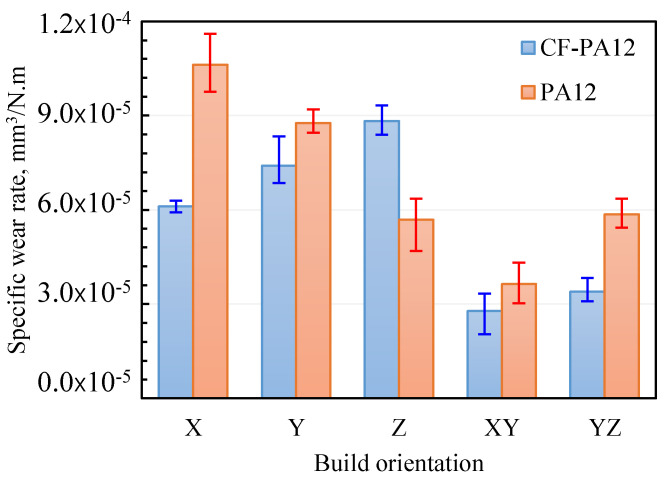
Specific wear rates of CF-PA12 and pure PA12.

After experiments, the wear volume is measured for the purpose of calculating the specific wear rate as its inverse identifies the wear resistance of the material. The specific wear rate (*K_s_*) is calculated as [27]:

(1) $$K_{s}=\frac{∆V}{F_{N}\times L}$$

where Δ*V*, *F_N_*, and *L* represent wear volume in (mm^3^), normal load (N), and sliding distance (m), respectively. Figure 7 shows a comparison between the specific wear rates of CF-PA12 and pure PA12 specimens along the different orientations. It is apparent from Figure 7 that, except for the Z-oriented specimen, all CF-PA12 specimens have higher wear resistance than those of pure PA12. Indeed, reinforcing laser-sintered PA12 with short carbon fibers increases the wear resistance of CF-PA12 composite by 15–40% depending on the build orientation. However, both materials have comparable wear resistance along the XY-plane. Unlike other build orientations, the higher wear rate of the Z-oriented CF-PA12 specimen is pertinent to the continuous removal of transfer film by the peeling action of protuberate carbon fibers. In addition, pronounced signs of adhesion were detected at the interface, which emphasized the hypothesis of continuous migration of PA12 onto the disc surface.

The results from this study suggest that the friction and wear characteristics of the CF-PA12 composite pertain to the interaction at the CFs/disc interface. Additionally, the alignment of CFs relative to sliding direction significantly affects the friction and wear patterns that dominate the tribo-surface.

As the friction and wear behavior of thermoplastic composites is very sensitive to sliding conditions, the abovementioned findings must be interpreted with caution since they describe the behavior under the specified experimental conditions and cannot be extrapolated for other conditions. To complete the picture, further experiments under different applied loads, sliding speeds, and sliding durations need to be carried out. Then, artificial intelligence (AI) can be applied to determine the proper build orientation for the manufacturing of functional parts with tailored tribological properties [16].

Taken together, the results from Figure 3, Figure 4, Figure 5, Figure 6 and Figure 7 suggest that where friction resistance is concerned, the bearing of CF-PA12 should be aligned perpendicular to X and Y orientations during the SLS of the functional part. On the other hand, where wear resistance is concerned, the tribo-surface of the CF-PA12 part must be aligned perpendicular to either the XY or YZ planes in the SLS build chamber. The results reported here can be beneficial in designing functional parts of the CF-PA12 composite for aerospace and automotive industries where the composite interacts with its steel counterpart.

### 3.5. Scanning Electron Microscopy (SEM)

**Figure 8:**
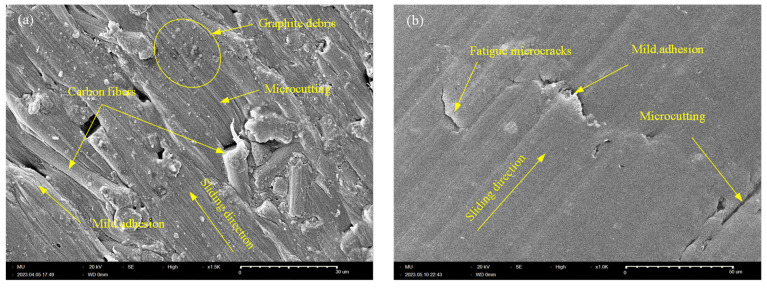
SEM of X-oriented specimen. (**a**) CF-PA12; (**b**) PA12.

Tribo-surface characterization using SEM is a reliable tool to obtain a clear insight into the surface/sub-surface tribological interactions. It also provides a means for predicting the wear patterns that dominate the tribo-surface. Figure 8 shows SEM of the worn surface of X-oriented CF-PA12 and pure PA12 specimens. The tribo-surface of CF-PA12 features obvious signs of microcutting grooves along with regions of mild adhesion. In addition, a close look at the worn surface can detect the existence of tiny graphite debris that results from direct CF/disc interactions. Furthermore, the presence of protruded CF further emphasizes its role in supporting a major part of the applied normal load. The results from Figure 8a suggest that abrasive wear predominates the tribo-surface in this case; however, this does not rule out the contribution of the existing mild adhesion to the frictional behavior of the specimen. Accordingly, the X-oriented CF-PA12 specimen has modest COF and, consequently, a low level of friction-induced noise. Meanwhile, Figure 8b shows obvious signs of surface fatigue microcracks normal to the sliding direction that result from repeated formation/breaking down of adhesive junctions. Additionally, signs of mild adhesion can be observed. However, the presence of deep microcutting grooves affirms the significant role of an abrasive component in the frictional resistance of the X-oriented pure PA12 specimen. As a result, the X-oriented PA12 specimen exhibits the lowest wear resistance among all tested samples.

**Figure 9:**
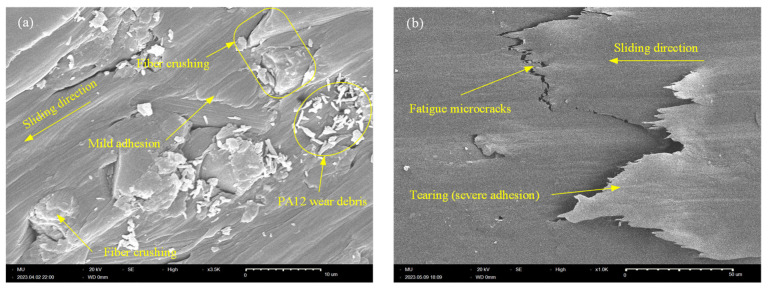
SEM of Y-oriented specimen, (**a**) CF-PA12, (**b**) PA12.

For Y-oriented CF-PA12 (see Figure 9a) the tribo-surface shows marks of mild adhesion combined with short rolls of PA12 wear debris. Another interesting feature of the Y-oriented CF-PA12 tribo-surface is the pulverized edges of carbon fibers, which is unmistakable evidence of fiber crushing by steel disc asperities. Fiber crushing results in the spreading of small-size irregular-shape graphite particles that act to lubricate the interface, thus reducing the COF, frictional noise, and interface temperature significantly (the COF, interface temperature, and friction-induced noise of Y-oriented CF-PA12 are the lowest among all specimens). In contrast to CF-PA12, the tribo-surface of the Y-oriented PA12 specimen shown in Figure 9b is characterized by clear marks of strong adhesion that are manifested by the appearance of long tongues in some regions and fatigue microcracks in others. This result suggests that adhesive wear dominates the tribo-surface of the Y-oriented pure PA12 specimen.

**Figure 10:**
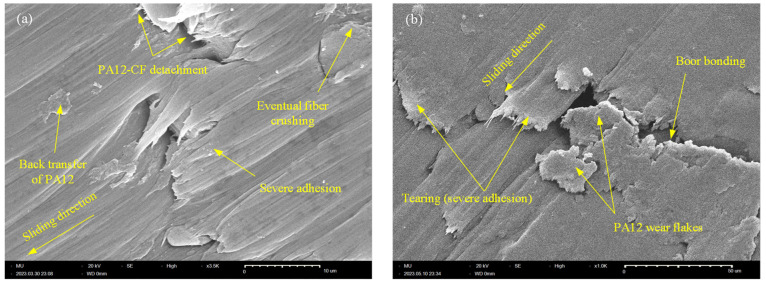
SEM of Z-oriented specimen. (**a**) CF-PA12; (**b**) PA12.

Figure 10 shows the SEM of Z-oriented CF-PA12 and pure PA12 specimens. As was explained earlier, Figure 10a further emphasizes that an adhesive wear pattern dominates the tribo-surface of CF-PA12, and this was the main reason behind the comparatively elevated levels of the COF, interface temperature, and frictional noise of the Z-oriented CF-PA12 specimen. Despite the observed good bonding of CF to the PA12 matrix, the strong adhesion of the PA12 matrix to the counter surface can cause CF/PA12 wall separation, which, in turn, facilitates the plastic flow of the matrix. In addition, the tribo-surface shows marks of PA12 back-transfer; this can be the result of the CF/disc interaction. Furthermore, CF pulverization can be noticed, which releases graphite powder at the interface and eventually improves the frictional behavior of the Z-oriented CF-PA12 specimen. Similarly, the results from Figure 10b depict that the adhesive wear pattern dominates the tribo-surface of the Z-oriented pure PA12 specimen. The worn surface shows clear marks of strong adhesion along with continuous microcutting grooves, which promote the adhesion resistance of PA12. Besides that, weakly attached PA12 flakes are observed on the worn surface; this back-transfer occurs if the transfer film does not stick firmly to the counter surface, and it was reported that the wear rate is influenced by the rate of film removal from the counter surface rather than by the rate of film transfer to the counter surface [19,28].

**Figure 11:**
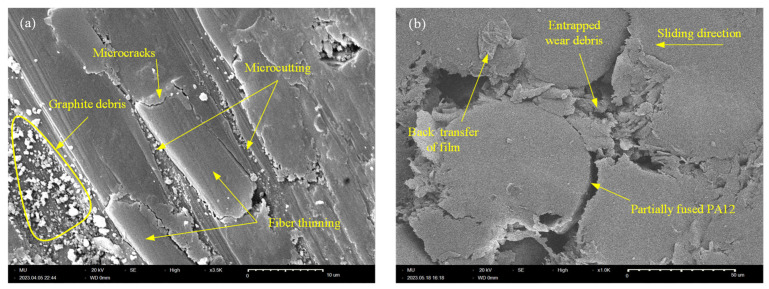
SEM of XY-plane specimen. (**a**) CF-PA12; (**b**) PA12.

Having discussed the characteristics of worn surfaces normal to the X-, Y-, and Z-axis, the following sections will analyze the features of worn surfaces of in-plane specimens (i.e., the specimens built along the XY-plane and YZ-plane). Figure 11 shows the SEM of the tribo-surface of XY-plane CF-PA12 and pure PA12 specimens. Unlike other CF-PA12 specimens, Figure 11a reveals that the dominant wear pattern of the XY-plane tribo-surface is abrasive wear; there are clear marks of discontinuous microcutting grooves along the sliding direction. It is worth noting that the visible surface microcracks are not adhesion-induced, rather they originate near CF boundaries at regions of poor bonding with PA12 matrix; upon coalescence of these microcracks, delamination of the PA12 matrix may occur [23]. Furthermore, closer inspection of the tribo-surface shows CF thinning as a result of the abrasion action, which produces fine graphite particles collected in deep surface cavities. As sliding continues, thin CF are pulled out of the surface, thus introducing the PA12 matrix into direct contact with the disc surface and, consequently, the COF as well as the friction noise increases significantly [29]. On the contrary, Figure 11b shows that adhesive wear predominates the worn surface of the XY-plane specimen of pure PA12. The surface is characterized by regions of partially sintered PA12 along with deep cavities between sintered regions at which wear debris is collected. Figure 11b reveals that there has been a steady increase in the real contact area and, as a consequence, the adhesion-prone area; this was the main reason behind the sluggish increase of both the COF and interface temperature of this specimen.

**Figure 12:**
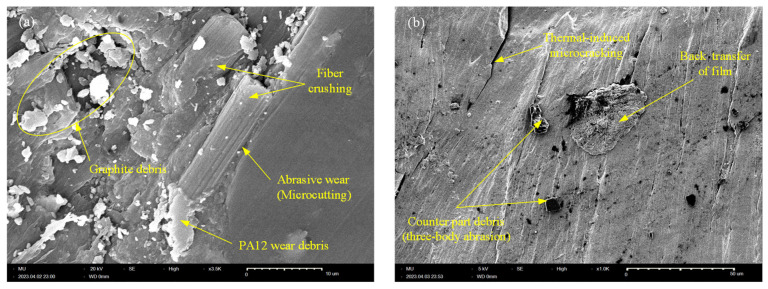
SEM of YZ-plane specimen. (**a**) CF-PA12; (**b**) PA12.4. Conclusions.

In an analogous manner to the XY-plane CF-PA12 specimen, Figure 12a shows that the worn surface of the YZ-oriented specimen experienced strong abrasive wear of both the matrix and the reinforcement; this appears in the form of pronounced fiber crushing along with lumps of PA12 debris. Abrasive wear ceases upon removal of soft PA12 regions and CF supports the majority of the applied load; however, this comes at the expense of frictional noise, which increases significantly in this event. In fact, reinforcing PA12 with CFs decreases the maximum elongation at break and, consequently, reduces the abrasive wear resistance of CF-PA12 composite [30]. On the other hand, the tribo-surface of the YZ-oriented pure PA12 specimen shows clear signs of thermal-induced microcracking along with back-transfer of PA12 film, which suggests that adhesive wear predominates the sliding surface.

This work was devoted to studying the effect of reinforcing the PA12 matrix with short carbon fibers on the friction and wear characteristics of specimens manufactured by the selective laser sintering (SLS) technique. The COF, approximate interface temperature, friction-induced noise, and specific wear rate were measured for CF-PA12 as well as the pure PA12 specimens that were built along five different orientations. Scanning electron microscopy (SEM) was used to investigate the wear patterns that predominated the tribo-surface. The results revealed that, in contrast to pure PA12, the COF of CF-PA12 specimens seem to be orientation-independent and the average steady-state COF of CF-PA12 is about 25% lower than that of pure PA12. Additionally, the results showed that the wear resistance of CF-PA12 composite is 15–40% higher than that of pure PA12 specimens, and the interface temperature of CF-PA12 is lower than that of pure PA12 along all build orientations.

Further, the SEM of tribo-surfaces revealed that adhesive wear is the dominant wear pattern of pure PA12 specimens, while adhesive and abrasive wear patterns can coexist side by side in CF-PA12 specimens. After a long sliding time, fiber crushing and thinning releases graphite debris at the interface, thus decreasing the COF and friction-induced noise. 

However, further experimental data under different loads, sliding speeds, and sliding durations can be integrated with a suitable artificial intelligence module to determine the proper build orientation for the manufacturing of functional parts with customized tribological characteristics. 

## Figures and Tables

**Table 1:** SLS fabrication parameters of CF-PA12 and pure PA12 specimens.

	PA2200 (PA12)	CF-PA12 (CarbonMide)
Powder	White color	Black color
SLS system	EOS P730 Machine	EOS P390 Machine
Outline power (W)	32.3	7.72
Outline scanning speed (mm/s)	3000	700
Hatching power (W)	38	10.8
Hatching speed (mm/s)	4500	1100
Scan spacing (mm)	0.3	0.2
Layer thickness (mm)	0.15	0.15

**Table 2:** Experimental conditions of the pin-on-disc tribometer.

Normal load (N)	50
Disc rotation speed (rpm)	120
Sliding track radius (mm)	20
Sliding speed (mm/s)	250
Test duration (min)	45
Disc initial temperature (°C)	29–30
Background noise level (dBA)	35–37
Humidity (%)	7–10

## Data Availability

The data are contained within the article.
